# Supplementary material for: Metabolite Fraction Libraries for Quantitative NMR Metabolomics
Source: bioRxiv. 2025 Dec 31:2025.12.30.696914. Preprint. [Version 1] doi: 10.64898/2025.12.30.696914 (PMC12776305; doi:10.64898/2025.12.30.696914)
Supplement: Supplement 1 [file NIHPP2025.12.30.696914v1-supplement-1.pdf]

bioRxiv preprint doi: https://doi.org/10.64898/2025.12.30.696914; this version posted December 31, 2025. The copyright holder for this preprint (which was not certified by peer review) is the author/funder, who has granted bioRxiv a license to display the preprint in perpetuity. It is made available under aCC-BY 4.0 International license.

Supplement: Metabolite Fraction Libraries for Quantitative NMR Metabolomics

Christopher Esselman[1,4], Kara Garrison[2,4], Leandro Ponce[3,4], Ricardo M. Borges[5], Frank Delaglio[6], Arthur S. Edison[1,2,4]

1. Institute of Bioinformatics, University of Georgia, Athens, Georgia, USA.
2. Department of Biochemistry and Molecular Biology, University of Georgia, Athens, Georgia, USA.
3. College of Engineering, University of Georgia, Athens, Georgia, USA.
4. Complex Carbohydrate Research Center, University of Georgia, Athens, Georgia, USA.
5. Instituto de Pesquisa de Produtos Naturais Walter Mors, Universidade Federal do Rio de Janeiro, Rio de Janeiro, Brazil
6. Institute for Bioscience and Biotechnology Research, National Institute of Standards and Technology and the University of Maryland, Rockville, Maryland, USA.

bioRxiv preprint doi: https://doi.org/10.64898/2025.12.30.696914; this version posted December 31, 2025. The copyright holder for this preprint (which was not certified by peer review) is the author/funder, who has granted bioRxiv a license to display the preprint in perpetuity. It is made available under aCC-BY 4.0 International license.

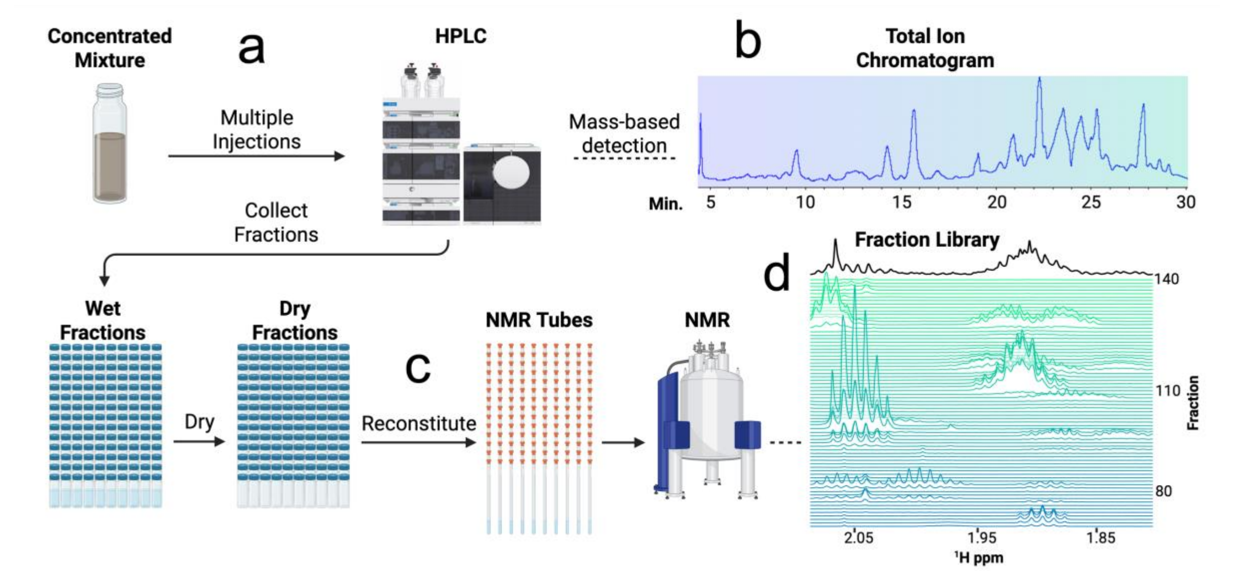

Figure Supplementary 1: Experimental workflow of creating a metabolite Fraction Library (mFL). **a**, A concentrated biological mixture is injected multiple times into an HPLC using the same fraction vials for each injection. **b**, If a mass spectrometer is coupled to the HPLC, a total ion chromatogram can be collected during fractionation. **c**, The fractions are then dried using a vacuum concentrator and reconstituted into NMR tubes. **d**, One-dimensional (1D) proton (^1H) NMR is collected for each fraction.

bioRxiv preprint doi: https://doi.org/10.64898/2025.12.30.696914; this version posted December 31, 2025. The copyright holder for this preprint (which was not certified by peer review) is the author/funder, who has granted bioRxiv a license to display the preprint in perpetuity. It is made available under aCC-BY 4.0 International license.

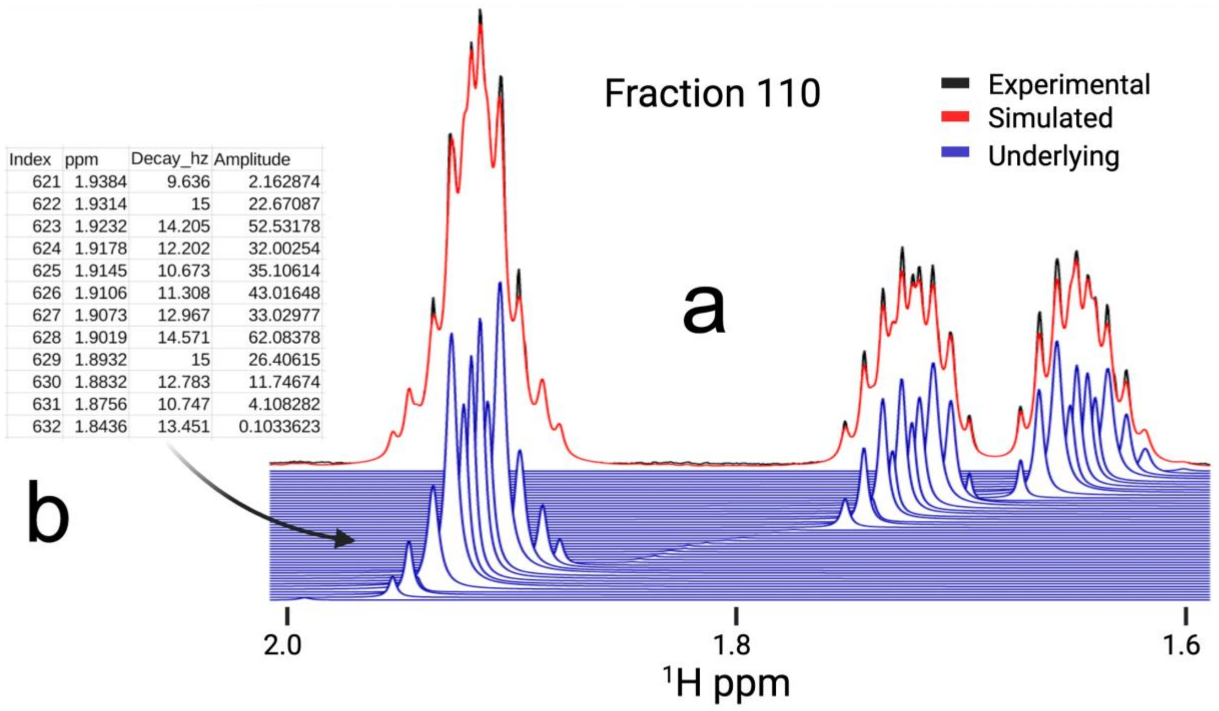

Figure Supplementary 2: Time domain modeling of NMR data via spectral automated NMR decomposition (SAND). **a**, The black spectrum is the original Fourier transformed (FT) data. The blue peaks are the frequency reconstructed underlying Lorentzian peaks time domain modeled by SAND. The red spectrum is the summation of the blue Lorentzian peaks. **b**, The SAND tabular domain data includes the frequency, decay, amplitude, and phase of every peak in the modeled spectrum. The phase is usually adjusted to zero before SAND.

bioRxiv preprint doi: https://doi.org/10.64898/2025.12.30.696914; this version posted December 31, 2025. The copyright holder for this preprint (which was not certified by peer review) is the author/funder, who has granted bioRxiv a license to display the preprint in perpetuity. It is made available under aCC-BY 4.0 International license.

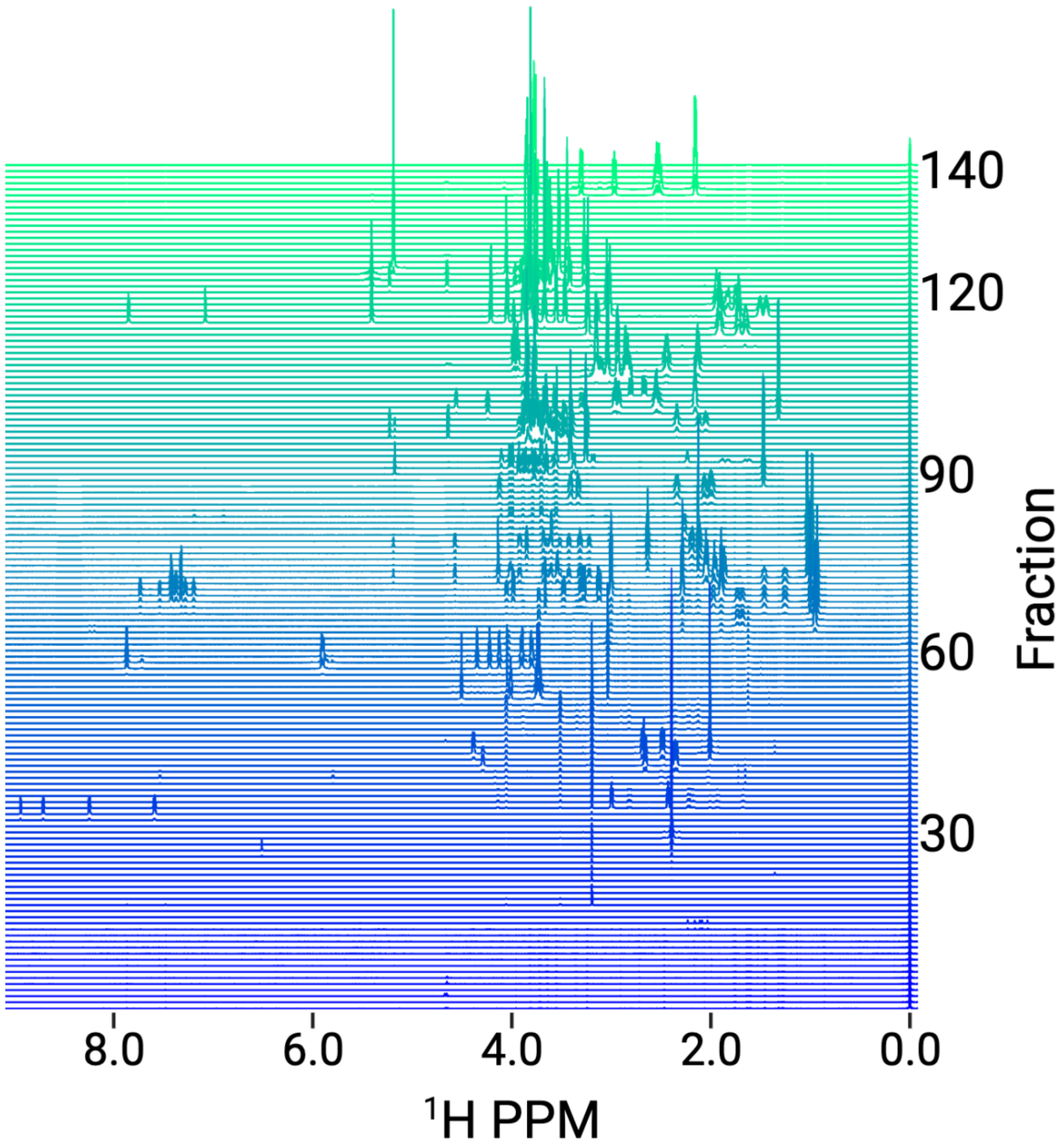

Figure Supplementary 3: Metabolite fraction library (mFL) of ground-truth mixture.

bioRxiv preprint doi: https://doi.org/10.64898/2025.12.30.696914; this version posted December 31, 2025. The copyright holder for this preprint (which was not certified by peer review) is the author/funder, who has granted bioRxiv a license to display the preprint in perpetuity. It is made available under aCC-BY 4.0 International license.

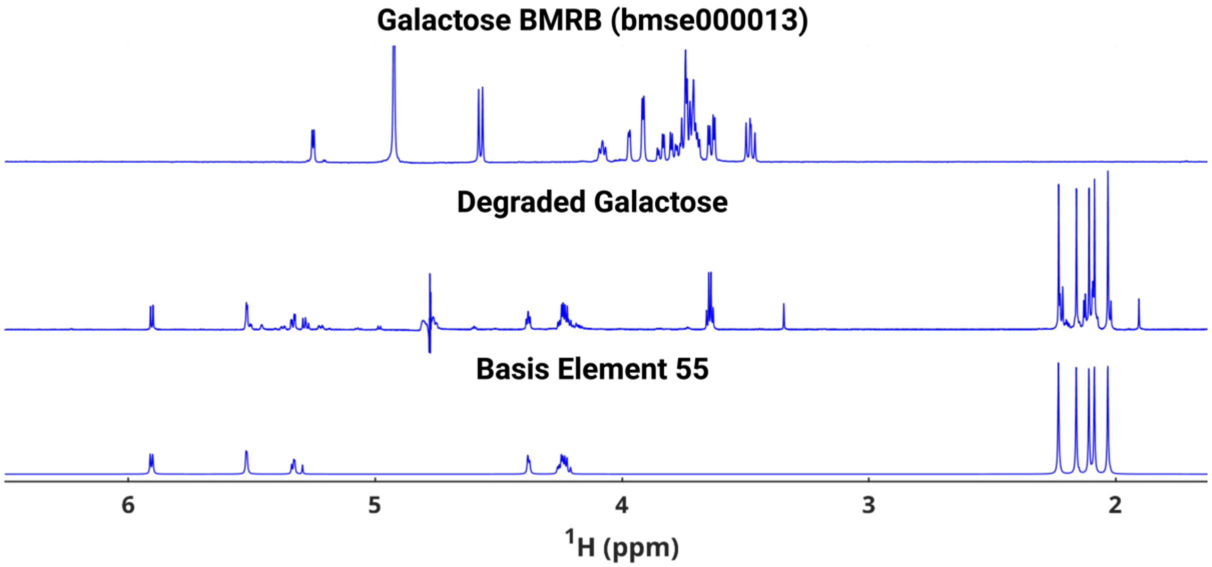

Figure Supplementary 4: BMRB reference spectrum (bmse000013) of galactose, the ground-truth spectrum of the degraded galactose sample, and metabolite basis element 55. Unexpectedly, the galactose obtained for this study does not match the known spectrum of galactose from the BMRB database. The peaks in the galactose sample that are not captured in basis element 55 may be peaks of another contaminant.

bioRxiv preprint doi: https://doi.org/10.64898/2025.12.30.696914; this version posted December 31, 2025. The copyright holder for this preprint (which was not certified by peer review) is the author/funder, who has granted bioRxiv a license to display the preprint in perpetuity. It is made available under aCC-BY 4.0 International license.

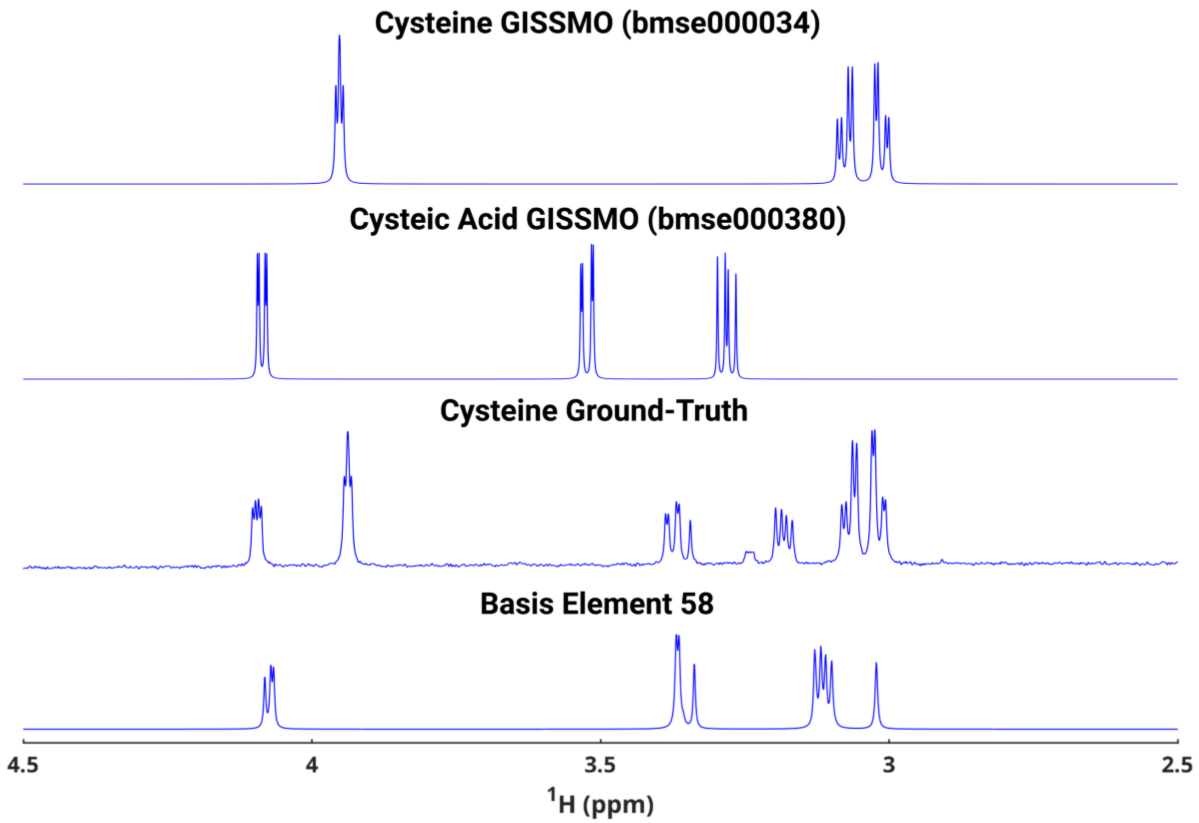

Figure Supplementary 5: GISSMO reference spectrum (bmse000034) of cysteine, GISSMO reference spectrum (bmse000380) of cysteic acid, the ground-truth spectrum of the cysteine sample, and metabolite basis element 58. The cysteine ground-truth sample exhibits resonances that resemble those of cysteine and its oxidation product, cysteic acid. Changes in chemical shift are likely due to variations in pH. We quantified cysteic acid resonances in basis element 58.

bioRxiv preprint doi: https://doi.org/10.64898/2025.12.30.696914; this version posted December 31, 2025. The copyright holder for this preprint (which was not certified by peer review) is the author/funder, who has granted bioRxiv a license to display the preprint in perpetuity. It is made available under aCC-BY 4.0 International license.

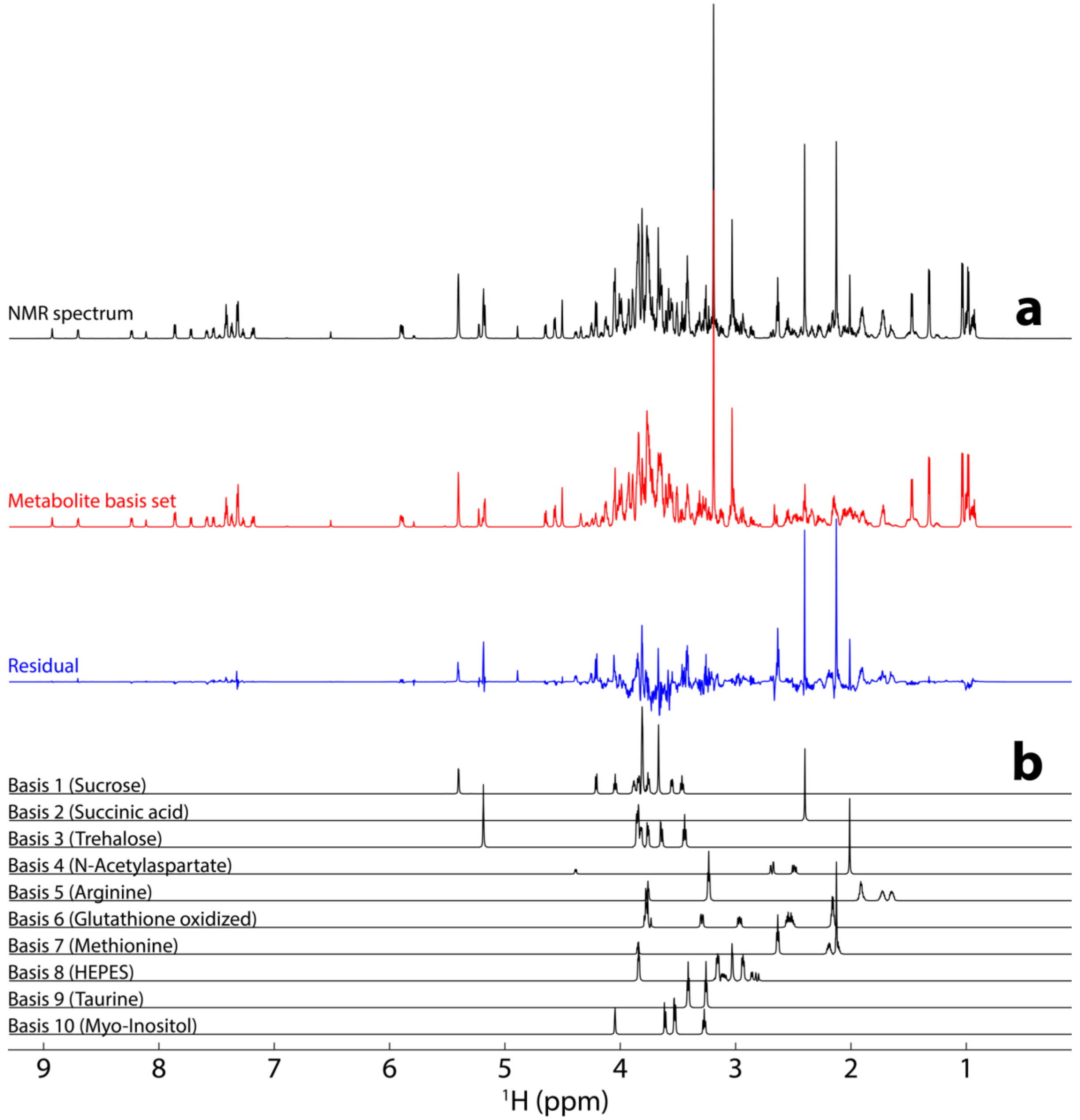

Figure Supplementary 6: a, BATMAN fit of the mBS from our ground-truth set of 53 synthetic metabolite solutions with the first 10 mBS elements excluded from fitting. The NMR spectrum at the top (black) is one of ten ground-truth experimental mixtures for this study, and the same spectrum is shown in Figure 5. The metabolite basis set (mBS) shown in red is the BATMAN fit. The blue trace shows the residuals (wavelet fit) from the Bayesian analysis. **b,** Spectra of the first 10 basis set elements excluded from the fit. In this example, the quantification model accounts for 82% of the total spectral intensity.

bioRxiv preprint doi: https://doi.org/10.64898/2025.12.30.696914; this version posted December 31, 2025. The copyright holder for this preprint (which was not certified by peer review) is the author/funder, who has granted bioRxiv a license to display the preprint in perpetuity. It is made available under aCC-BY 4.0 International license.

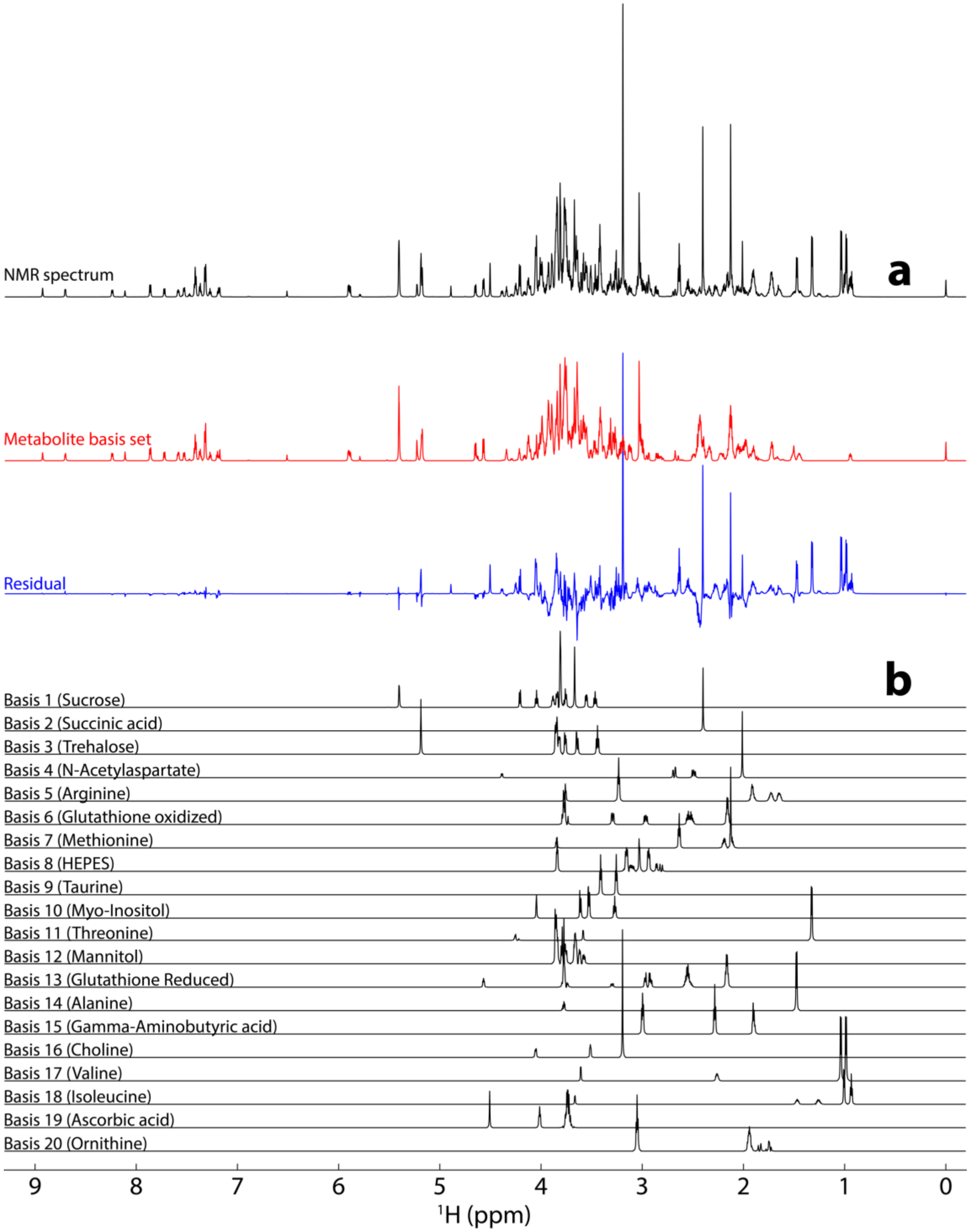

Figure Supplementary 7: a, BATMAN fit of the mBS from our ground-truth set of 53 synthetic metabolite solutions with the first 20 mBS elements excluded from fitting. The NMR spectrum at the top (black) is one of ten ground-truth experimental mixtures for this study, and the same spectrum is shown in Figure 5. The metabolite basis set (mBS) shown in red is the BATMAN fit. The blue trace shows the residuals (wavelet fit) from the Bayesian analysis. **b,** Spectra of the first 20 basis set elements excluded from the fit. In this example, the quantification model accounts for 56% of the total spectral intensity.

bioRxiv preprint doi: https://doi.org/10.64898/2025.12.30.696914; this version posted December 31, 2025. The copyright holder for this preprint (which was not certified by peer review) is the author/funder, who has granted bioRxiv a license to display the preprint in perpetuity. It is made available under aCC-BY 4.0 International license.

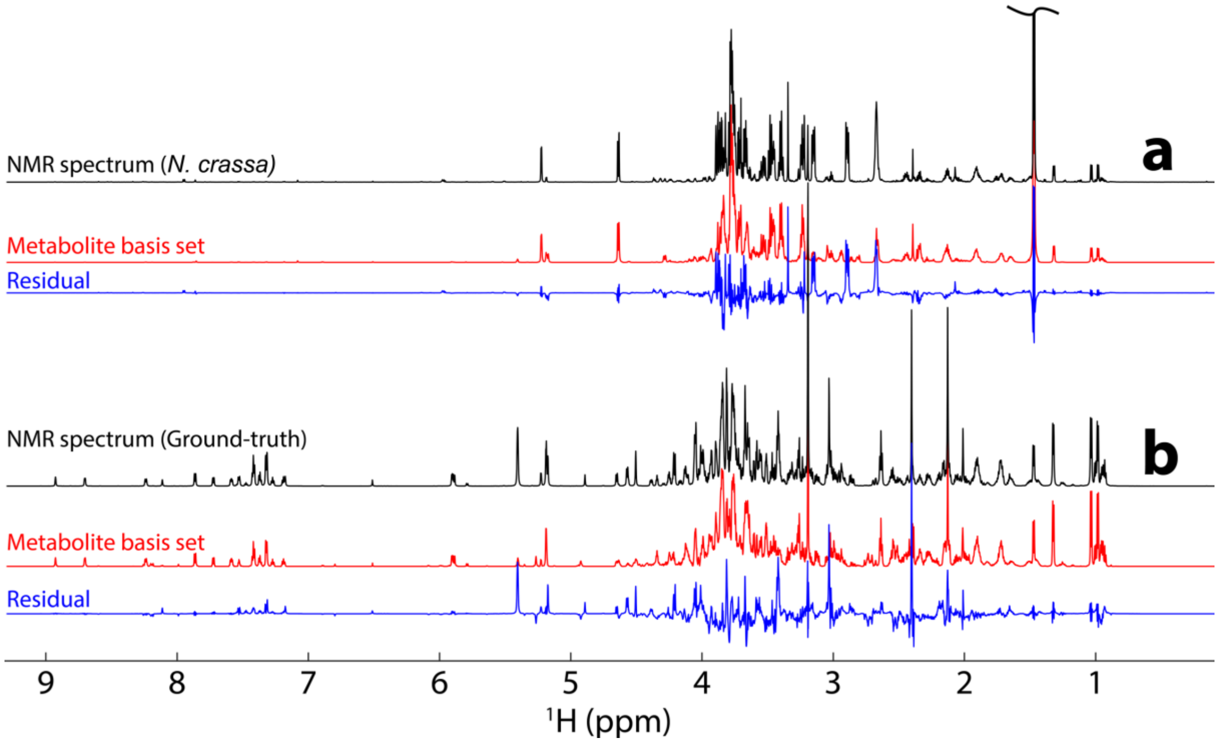

Figure Supplementary 8: a, BATMAN fit of the mBS from the ground-truth mFL to the *N. crassa* unfractionated mixture spectrum. The BATMAN fit explained 83% of the original spectral intensity. **b,** BATMAN fit of the mBS from the *N. crassa* mFL to one of the ground-truth mixture spectra. In this example, the quantification model accounts for 82% of the total spectral intensity.

bioRxiv preprint doi: https://doi.org/10.64898/2025.12.30.696914; this version posted December 31, 2025. The copyright holder for this preprint (which was not certified by peer review) is the author/funder, who has granted bioRxiv a license to display the preprint in perpetuity. It is made available under aCC-BY 4.0 International license.

Ground-Truth Methods

Creating Ground-Truth Spectra

The compounds used to create the single-component ground-truth reference spectra were common metabolites readily available in the laboratory. The milligram amount required for all 53 metabolites was calculated to prepare a 5 mL solution at a 100 mM concentration in H_2O. An approximate amount of powder was weighed for each metabolite and added to a 10 mL glass centrifugal tube. LC/MS grade H_2O was added, the tubes were vortexed, and stored at -20 ˚C. Due to solubility issues, not all metabolites fully dissolved in the solution.

Preparing Fractionation Mixture

10 µL was taken from each single-metabolite reference sample except for aspartate, in which 20 µL was taken and added to a 2 mL screw cap centrifuge tube. The tube was dried in a Centrivap and resuspended in 400 µL of 80:20 LC/MS grade MeOH: H_2O.

HPLC Fractionation

The fraction library was produced by three 100 µL injections using an Agilent 1260 Infinity HPLC with XBridge BEH Amide OBD Prep Column, 130 Å, 5 µm, 10 x 250 mm HILIC column at 25°C. Full scan data was collected using an Agilent Infinity Lab Single Quadrupole MSD in positive ion mode (50-1250 Da). OpenLab ChemStation software was used for data acquisition and visualization.

A 38 min linear gradient of 0.1% formic acid in H_2O (A) and 0.1% formic acid in ACN (B) was used for the fractionation. From 0-20 min, a linear gradient of 5% to 30% A was used, followed by a linear gradient of 30% to 50% A from 20-30 min, all at a flow rate of 3.5 mL/min. From 30-35 min, a linear gradient of 50% to 65% A was used, followed by an isocratic hold from 35-38 min, both at a flow rate of 2 mL/min. A post-time of 8 min was set to allow the system to equilibrate to the initial condition of 5% A before further injections. Between 0.9 and 30 min, 140 equally spaced fractions were collected, approximately 12.5 s per fraction. Fractionation was done over identical fraction vials for all three injections. Between injections 2 and 3 and after injection 3, the vials were dried using a Centrivap. The dried fraction vials were stored at -80 °C before NMR data collection.

Creation of Mixtures for Fitting

Ten mixtures of all metabolites were created. A random number generator was used to generate values between 1-55 µL to decide how much of each single-compound ground-truth sample would be added to each mixture (Table Supplementary 4). The mixtures were then dried using a Centrivap and reconstituted in 550 µL of buffered D_2O (100 mM sodium phosphate buffer and 0.333 mM DSS-D_6 at 7.4 pH). 55 µL of the solutions were pipetted into 1.7 mm Bruker SampleJet NMR tubes.

bioRxiv preprint doi: https://doi.org/10.64898/2025.12.30.696914; this version posted December 31, 2025. The copyright holder for this preprint (which was not certified by peer review) is the author/funder, who has granted bioRxiv a license to display the preprint in perpetuity. It is made available under aCC-BY 4.0 International license.

NMR Data Acquisition

The fractions were reconstituted in 55 µL D_2O buffer and transferred to 1.7 mm Bruker SampleJet tubes. Solvent blanks were placed in positions 1, 96, 97, and 144 amongst the fractions. NMR data were collected using a Bruker Avance Neo console on an Oxford 800 MHz magnet with a 1.7 mm TCI cryoprobe and a cooled SampleJet sample changer. One-dimensional NMR data were acquired at 298K using a "noesypr1d" pulse sequence, and 32,768 points were collected with 8 dummy scans and 64 scans for each sample. The ten mixtures were run on the same instrument. The data were acquired at 298K using a "noesypr1d" pulse sequence, and 32,768 points were collected with 4 dummy scans and 16 scans for each sample. The data were automatically updated to NAN and NMRbox for processing.

Data processing and SAND

Prior to applying SAND, the spectra for ground-truth reference samples and mixtures were processed using the NMRPipe batch processing scheme, and reference deconvolution was applied using DSS as the reference lineshape, with a target linewidth of 1 Hz and a line broadening of 1.5 Hz. The reference samples were then time-domain modeled by SAND over the range of 9.1 ppm to -0.15 ppm.

Relative Concentrations of Basis Set Elements

Because of the limited solubility of some compounds, the expected concentrations do not always match the actual concentrations of the metabolites in the mixtures. To account for this, metabolite concentrations in the mixtures were determined by comparing the integral of isolated resonances to the integral of DSS using Mnova software. The newly calculated concentrations and the BATMAN relative concentrations were then compared in MATLAB.

bioRxiv preprint doi: https://doi.org/10.64898/2025.12.30.696914; this version posted December 31, 2025. The copyright holder for this preprint (which was not certified by peer review) is the author/funder, who has granted bioRxiv a license to display the preprint in perpetuity. It is made available under aCC-BY 4.0 International license.

Basis	Metabolite	Confidence
1	Trehalose	High
2	Arabitol	Medium
3	HEPES	Medium
4	Alanine	High
5	Acetylcholine	Medium
6	Valine	High
7	Glutamic Acid	High
8	Succinic Acid	High
9	Glycine	High
10	Arginine	High
11	Glucose	High
12	Lactate	High
13	Glycerol	High
14	Choline	High
15	Trimethylamine-N-oxide	High
16	Threonine	High
17	Glutamine	High
18	Glutathione Reduced	Medium
19	arginine_glutamate	Medium
20	5_acetylamido_2_chloroaniline/Chlorogenic acid	Medium
21	Lysine	High
22	betaine	Medium
23	carnitine	Medium
24	uk(1)	N/A
25	Ornithine	High
26	Formaldehyde	Medium
27	uk(2)	N/A
28	Dihydrouracil	Medium
29	Ethanolamine	High
30	Xylonate	Medium
31	uk(3)	N/A
32	Leucine	High
33	2_aminoadipic acid	Medium
34	uk(4)	N/A
35	GABA	High
36	Histidine	High
37	Serine	High
38	Citrulline	Medium
39	Malic Acid	High
40	uk(5)	N/A

bioRxiv preprint doi: https://doi.org/10.64898/2025.12.30.696914; this version posted December 31, 2025. The copyright holder for this preprint (which was not certified by peer review) is the author/funder, who has granted bioRxiv a license to display the preprint in perpetuity. It is made available under aCC-BY 4.0 International license.

41	Asparagine	High
42	Proline	High
43	Trimethylamine	Medium
44	uk(6)	N/A
45	Glutathione Oxidized	High
46	Mannitol	Medium
47	Aspartate	High
48	Hypotaurine	High
49	uk(7)	N/A
50	Isoleucine	High
51	Aminobutyric acid	High
52	AMP	High
53	Methyl-oxobutanoic-acid	High
54	Myo-inositol	Medium
55	uk(8)	N/A
56	uk(9)	N/A
57	Inosine	High
58	uk(10)	N/A
59	uk(11)	N/A
60	uk(12)	N/A
61	uk(13)	N/A
62	Pantothenate	High
63	Carbamyl_glutamate	Medium
64	Thymidine	Medium
65	UTP	Medium
66	alpha_glycerol_phosphate	High
67	uk(14)	N/A
68	Ethanolamine	High
69	uk(15)	N/A
70	uk(16)	N/A
71	Akg	High
72	acetyl_mannosamine	Medium
73	3_4_Dihydroxy_L_phenylalanine	Medium
74	uk(17)	N/A
75	phenylalanine	High
76	Adenosyl_homocysteine	Medium
77	melibiose	Medium
78	xanthosine/guanosine	Medium
79	uk(18)	N/A
80	acetyl_glucosamine_6_phosphate	Medium
81	uk(19)	N/A

bioRxiv preprint doi: https://doi.org/10.64898/2025.12.30.696914; this version posted December 31, 2025. The copyright holder for this preprint (which was not certified by peer review) is the author/funder, who has granted bioRxiv a license to display the preprint in perpetuity. It is made available under aCC-BY 4.0 International license.

82	carnitine	Medium
83	uk(20)	N/A
84	uk(21)	N/A
85	uk(22)	N/A
86	uk(23)	N/A
87	uk(24)	N/A
88	Uridine	High
89	NADPH	Medium
90	Succinic Acid	High
91	Sarcosine	Medium
92	Citrate	High
93	3-hydroxybutyrate	Medium
94	uk(25)	N/A
95	UMP	High
96	glucose-6-phosphate	Medium
97	Adenosyl_homocysteine	Medium
98	Adenosyl_homocysteine	Medium
99	Trimethylamine	Medium
100	Fructose-6-phosphate	Medium
101	uk(26)	N/A
102	Acetyl_glutamic_acid	Medium
103	uk(27)	N/A
104	methyl_histidine	Medium
105	carnitine	Medium
106	Biotin	Medium
107	betaine	Medium
108	uk(28)	N/A
109	acetyl_aspartic_acid	Medium
110	Glucosamine	Medium
111	pyridoxamine_5_phosphate	Medium
112	uk(29)	N/A
113	uk(30)	N/A
114	Threitol	High
115	uk(31)	N/A
116	Nicotinamide	High
117	niacin	High
118	tryptophan	High
119	uk(32)	N/A
120	uk(33)	N/A
121	gamma_galactone	Medium
122	uk(34)	N/A

bioRxiv preprint doi: https://doi.org/10.64898/2025.12.30.696914; this version posted December 31, 2025. The copyright holder for this preprint (which was not certified by peer review) is the author/funder, who has granted bioRxiv a license to display the preprint in perpetuity. It is made available under aCC-BY 4.0 International license.

123	uk(35)	N/A
124	xylitol	Medium
125	carnitine	Medium
126	uk(36)	N/A

bioRxiv preprint doi: https://doi.org/10.64898/2025.12.30.696914; this version posted December 31, 2025. The copyright holder for this preprint (which was not certified by peer review) is the author/funder, who has granted bioRxiv a license to display the preprint in perpetuity. It is made available under aCC-BY 4.0 International license.

**Metabolite**	**Basis**
2-aminoadipic acid	Basis 46
Adenine	Basis 60
Alanine	Basis 14
AKG	Basis 41
Arginine	Basis 5
Ascorbic acid	Basis 19
Asparagine	Basis 39
Aspartate	Basis 37
Benzoic acid	Basis 59
Choline	Basis 16
Creatinine	Basis 25
Cysteine	Basis 58*
Fructose	Basis 38
Fumaric acid	Basis 47
GABA	Basis 15
Galactose	Basis 55*
Glucose	Basis 36
Glutamic Acid	Basis 40
Glutamine	Basis 30
Glutathione Oxidized	Basis 6
Glutathione Reduced	Basis 13
Glycine	Basis 22
HEPES	Basis 8
Histidine	Basis 29
Isoleucine	Basis 18
Leucine	Basis 26
Lysine	Basis 21 + Basis 34
Malic Acid	Basis 33
Maltose	Basis 23
Mannitol	Basis 12
Mannose	Basis 35
Methionine	Basis 7
myo-Inositol	Basis 10
N-acetyl Aspartic acid	Basis 4
Nicotinamide	Basis 45
Ornithine	Basis 20 + Basis 32
Phenylalanine	Basis 28
Proline	Basis 42
Pyruvate	
Quinic Acid	Basis 24

bioRxiv preprint doi: https://doi.org/10.64898/2025.12.30.696914; this version posted December 31, 2025. The copyright holder for this preprint (which was not certified by peer review) is the author/funder, who has granted bioRxiv a license to display the preprint in perpetuity. It is made available under aCC-BY 4.0 International license.

Serine	Basis 27
Sorbitol	Basis 49
Succinic acid	Basis 2
Sucrose	Basis 1
Taurine	Basis 9
Threonine	Basis 11
Trehalose	Basis 3
Tryptophan	Basis 44
Tyrosine	Basis 57
Uracil	Basis 56
Uridine	Basis 31
Valine	Basis 17
Xylose	Basis 43

bioRxiv preprint doi: https://doi.org/10.64898/2025.12.30.696914; this version posted December 31, 2025. The copyright holder for this preprint (which was not certified by peer review) is the author/funder, who has granted bioRxiv a license to display the preprint in perpetuity. It is made available under aCC-BY 4.0 International license.

Basis	Conc. (mM)
1	0.529
2	6.066
3	9.239
4	7.887
5	0.033
6	0.742
7	0.81
8	0.121
9	0.115
10	1.549
11	12.111
12	0.203
13	0.493
14	0.681
15	0.792
16	0.241
17	1.202
18	0.312
19	1.264
20	0.092
21	1.063
22	0.12
23	0.53
24	0.256
25	0.334
26	0.074
27	0.21
28	0.066
29	0.068
30	0.244
31	0.016
32	0.196
33	0.163
34	0.01
35	0.158
36	0.172
37	0.352
38	0.257
39	0.244
40	0.065

bioRxiv preprint doi: https://doi.org/10.64898/2025.12.30.696914; this version posted December 31, 2025. The copyright holder for this preprint (which was not certified by peer review) is the author/funder, who has granted bioRxiv a license to display the preprint in perpetuity. It is made available under aCC-BY 4.0 International license.

41	0.055
42	0.082
43	0.047
44	0.29
45	0.347
46	0.867
47	0.068
48	0.042
49	0.017
50	0.134
51	0.052
52	0.036
53	0.039
54	0.101
55	0.146
56	0.029
57	0.054
58	0.483
59	0.196
60	0.239
61	0.01
62	0.016
63	0.198
64	0.039
65	0.126
66	0.022
67	0.192
68	0.051
69	0.069
70	0.097
71	0.067
72	0.279
73	0.033
74	0.055
75	0.044
76	0.076
77	0.002
78	0.016
79	0.029
80	0.723
81	0.299

bioRxiv preprint doi: https://doi.org/10.64898/2025.12.30.696914; this version posted December 31, 2025. The copyright holder for this preprint (which was not certified by peer review) is the author/funder, who has granted bioRxiv a license to display the preprint in perpetuity. It is made available under aCC-BY 4.0 International license.

82	0.01
83	0.094
84	0.051
85	0.014
86	0.006
87	0.015
88	0.02
89	0.08
90	0.085
91	0.003
92	0.033
93	0.09
94	0.392
95	0.355
96	0.984
97	0.062
98	0.004
99	0.001
100	0.282
101	0.004
102	0.038
103	0.046
104	0.012
105	0.018
106	0.004
107	0.036
108	0.039
109	0.056
110	0.358
111	0.018
112	0.02
113	0.041
114	0.197
115	0.168
116	0.016
117	0.005
118	0.01
119	0.004
120	0.002
121	0.211
122	0.002

bioRxiv preprint doi: https://doi.org/10.64898/2025.12.30.696914; this version posted December 31, 2025. The copyright holder for this preprint (which was not certified by peer review) is the author/funder, who has granted bioRxiv a license to display the preprint in perpetuity. It is made available under aCC-BY 4.0 International license.

123	0.002
124	0.511
125	0.005
126	0.017
127	0.333

bioRxiv preprint doi: https://doi.org/10.64898/2025.12.30.696914; this version posted December 31, 2025. The copyright holder for this preprint (which was not certified by peer review) is the author/funder, who has granted bioRxiv a license to display the preprint in perpetuity. It is made available under aCC-BY 4.0 International license.

	Mixture 1	Mixture 2	Mixture 3	Mixture 4	Mixture 5	Mixture 6
Ornithine	51	51	2	21	41	46
Asparagine	7	39	5	43	25	50
Glycine	54	37	15	3	28	6
Choline	18	18	5	44	9	30
Histidine	37	33	19	14	3	14
Taurine	28	17	14	50	12	2
Maltose	48	43	34	54	35	2
Fumaric acid	5	29	34	52	45	48
Glutathione Oxidized	4	20	31	34	15	34
Sorbitol	43	12	20	31	4	4
Uridine	26	6	45	20	27	46
Valine	52	36	45	40	12	45
Adenine	21	32	4	10	4	39
Serine	15	22	19	18	50	20
2-aminoadipic acid	20	14	13	26	27	43
Quinic acid	3	25	24	12	4	31
Leucine	15	35	4	11	54	13
Malic acid	35	42	28	N/A	26	24
Creatinine	46	48	41	37	30	1
Proline	3	37	3	32	21	42
Sucrose	15	28	17	53	25	8
Methionine	43	47	10	55	54	44
Glutamic acid	43	2	39	17	18	44
Glucose	19	14	40	4	49	50
Lysine	13	19	31	39	28	12
HEPES	13	54	6	23	50	6
Ascorbic acid	6	43	34	47	31	7
Alanine	1	25	42	22	55	23
Isoleucine	43	41	12	16	54	25
AKG	29	37	4	23	48	24
Arginine	47	52	23	45	21	28
Trehalose	48	25	48	31	54	15
Xylose	13	19	16	39	32	52
Aspartate	22	46	29	2	55	10
Uracil	44	18	23	51	27	24
N-acetyl Aspartic acid	24	30	52	19	6	46
GABA	39	17	17	7	47	10
Myo-Inositol	3	46	20	7	48	31
Tyrosine	54	40	39	29	14	36
Pyruvate	30	2	14	19	54	23

bioRxiv preprint doi: https://doi.org/10.64898/2025.12.30.696914; this version posted December 31, 2025. The copyright holder for this preprint (which was not certified by peer review) is the author/funder, who has granted bioRxiv a license to display the preprint in perpetuity. It is made available under aCC-BY 4.0 International license.

Cysteine	32	15	34	24	26	46
Succinic acid	12	49	35	45	5	18
Nicotinamide	43	23	45	21	33	55
Tryptophan	35	31	47	40	31	7
Threonine	21	22	15	43	54	30
Benzoic acid	18	43	6	30	33	9
Glutathione Reduced	54	29	17	48	50	6
Fructose	18	31	55	26	31	22
Galactose	39	4	37	4	35	51
Glutamine	20	26	42	9	35	46
Mannitol	4	53	53	28	18	42
Mannose	3	29	52	54	23	37
Phenylalanine	8	28	21	35	37	11

bioRxiv preprint doi: https://doi.org/10.64898/2025.12.30.696914; this version posted December 31, 2025. The copyright holder for this preprint (which was not certified by peer review) is the author/funder, who has granted bioRxiv a license to display the preprint in perpetuity. It is made available under aCC-BY 4.0 International license.

Mixture 7	Mixture 8	Mixture 9	Mixture 10 (μL)
53	24	18	16
46	46	19	20
29	40	25	42
2	54	3	18
27	15	7	3
31	4	19	2
35	53	39	23
20	24	38	12
34	41	10	35
37	1	37	48
26	42	29	45
36	13	19	32
45	3	45	15
53	25	33	35
2	8	55	15
41	10	4	43
7	5	3	15
35	15	45	53
47	5	53	50
34	48	20	46
10	31	30	36
41	50	40	17
11	34	15	4
35	3	52	50
18	17	8	6
39	46	32	36
29	18	33	17
25	38	23	29
45	52	47	40
29	46	41	14
23	51	19	21
52	51	32	10
2	21	52	37
22	8	54	4
25	55	7	18
46	18	26	12
50	18	14	20
15	39	47	41
14	19	7	7
51	27	14	9

bioRxiv preprint doi: https://doi.org/10.64898/2025.12.30.696914; this version posted December 31, 2025. The copyright holder for this preprint (which was not certified by peer review) is the author/funder, who has granted bioRxiv a license to display the preprint in perpetuity. It is made available under aCC-BY 4.0 International license.

45	52	45	8
41	32	51	37
51	7	38	47
7	24	45	26
11	27	45	46
39	49	14	42
46	23	28	40
11	20	8	3
27	29	43	30
32	35	18	30
6	52	13	52
14	54	14	51
55	13	50	15
